# Case Report: Multi organ dysfunction in a dog following massive paper wasp (*Polistes rothneyi*) envenomation

**DOI:** 10.3389/fvets.2025.1558937

**Published:** 2025-03-11

**Authors:** Jeong-Min Lee, Seung-Keun Lee, Sun-Tae Lee

**Affiliations:** Korea Animal Medical Center, Cheongju, Republic of Korea

**Keywords:** acute kidney injury, canine, continuous renal replacement therapy, paper wasp, *Polistes rothneyi*

## Abstract

Paper wasp (*Polistes rothneyi*) envenomation is an emerging public threat in Asian countries, but its clinical manifestations are limited in veterinary medicine. A 2-year-old male Yorkshire Terrier was evaluated for symptoms including vomiting, melena, and anuria, and upon presentation, exhibited lethargy and signs of jaundice. The dog had a history of being stung multiple times by paper wasps (*Polistes rothneyi*) 3 days prior to admission. On blood examination, anemia, severe azotemia, hyperbilirubinemia, and significant elevation of creatine kinase were noted. Treatment was initiated with epinephrine, glucocorticoids, antihistamines, and fluid therapy. Despite these interventions, the condition worsened, necessitating the initiation of continuous renal replacement therapy. However, the dog subsequently developed bradycardia and hypotension, leading to cardiac arrest 48 h after presentation. This is the first case report describing the clinical manifestation of dogs envenomated by paper wasps (*Polistes rothneyi*). Massive paper wasp envenomation can cause multiple organ lesions, including renal, hepatic, and gastrointestinal damage.

## Introduction

The stings from members of the Hymenoptera order, including those from the Apoidea (bees), Vespoidea (wasps), and Formicidae (ants) families, present considerable potential health threats to dogs (1). This venom contains complex mixtures of allergenic proteins, active antigens, and peptides, which can lead to envenomation and potentially cause anaphylaxis (1). Hymenoptera stings are generally well tolerated and typically cause limited local reactions. However, in cases of massive envenomation, systemic reactions can occur, affecting multiple organs such as the kidneys, liver, nervous system, and lungs (2). Recently, wasps have successfully adapted to urban areas in many countries, leading to a significant increase in reported wasp stings, and they are now managed as a hazardous animal group (3). However, most reports on Hymenoptera stings in veterinary medicine focus on bee stings, with very limited documentation on wasp stings. The purpose of this report is to describe the clinical manifestations of a dog after massive paper wasp (*Polistes rothneyi*) envenomation.

## Case descriptions

**Figure 1:**
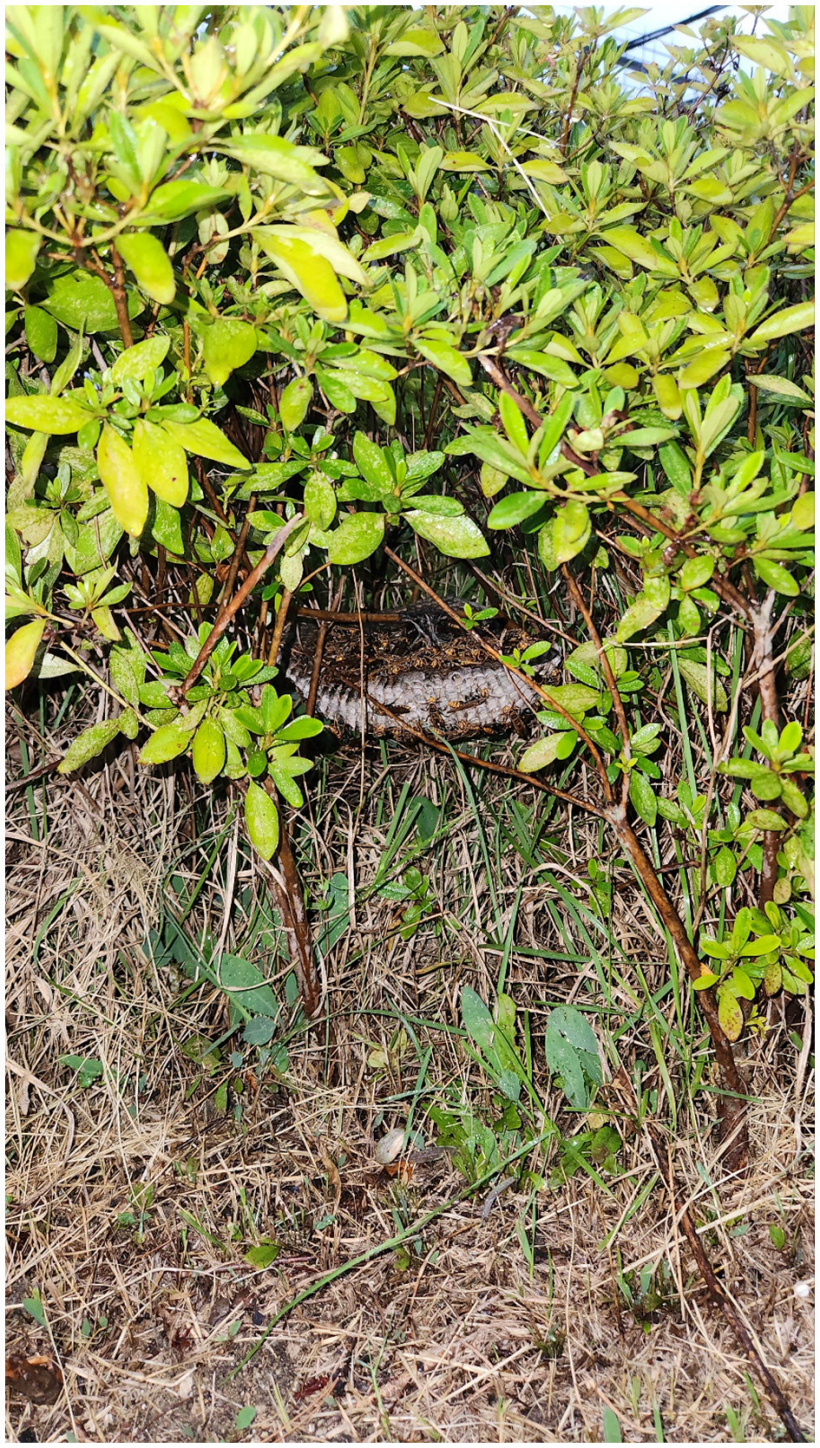
The nest of paper wasps (*Polistes rothneyi*).

A 2-year-old male Yorkshire Terrier weighing 4.88 kg referred to the emergency service for lethargy, vomiting, melena and anuria. The dog had a history of massive Hymenoptera envenomation, with over 100 stings, as reported by the owner, who directly witnessed the attack while the dog was urinating in the shrubs containing a nest 3 days before admission. The owner, who was also attacked by the Hymenoptera, called the emergency response center requesting nest removal. The nest was subsequently removed, and a taxonomist confirmed the Hymenoptera to be paper wasps (*Polistes rothneyi*) (Figure 1). The dog was reported to have had no prior health problems or medications before the envenomation and was fully vaccinated. The dog exhibited lethargy immediately after envenomation, followed by the onset of gastrointestinal signs, including vomiting and diarrhea, starting the next day. The condition progressively deteriorated, leading the owners to bring it to our hospital for further evaluation and treatment.

**Figure 2:**
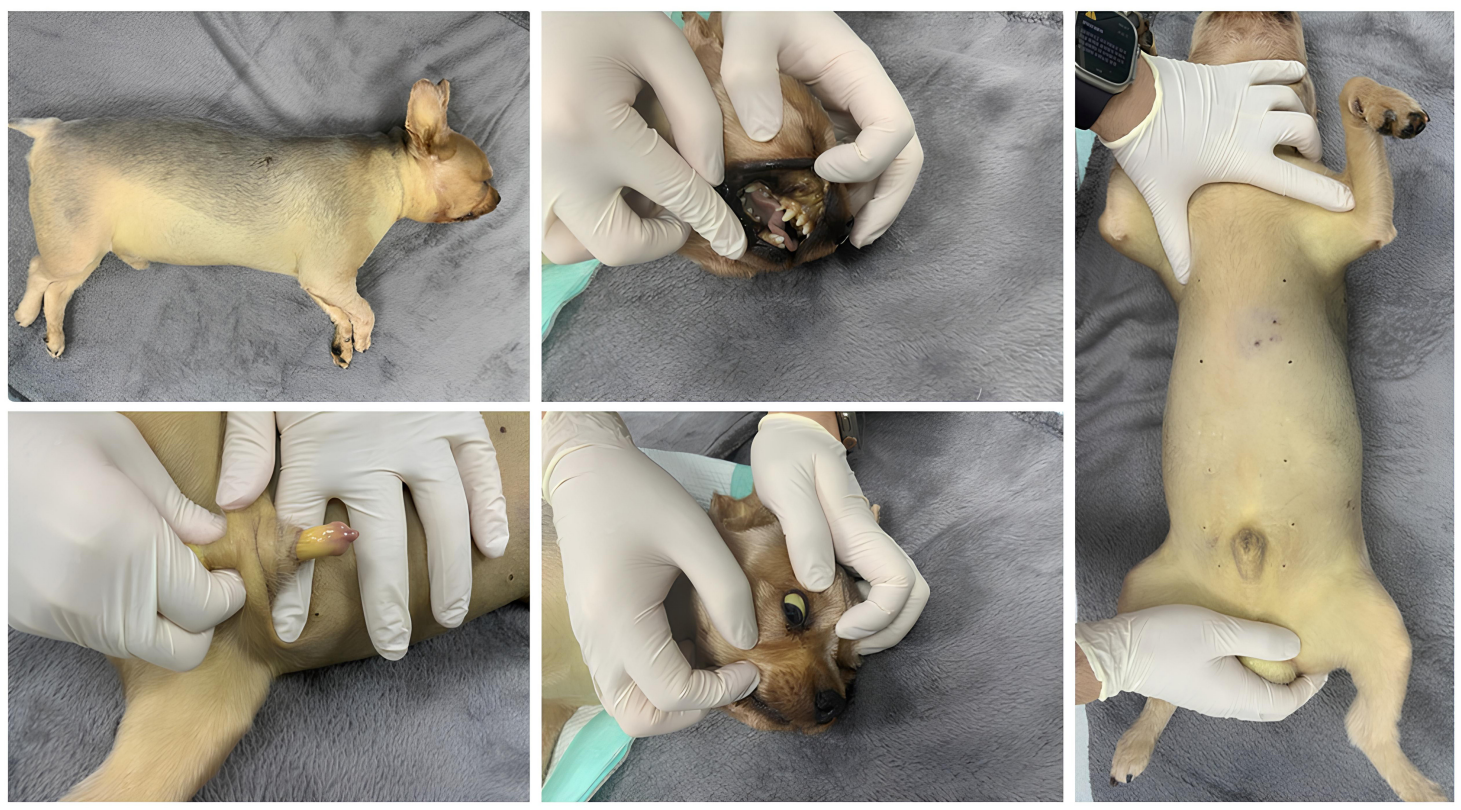
Yellow discoloration (jaundice) observed in the sclera, ears, and caudoventral abdomen of the paper wasp envenomated dog.

A general physical examination revealed facial swelling and jaundice, characterized by yellow discoloration throughout the body, including the ears, sclera, penis, and caudoventral abdomen (Figure 2). The patient was alert, auscultation was unremarkable, and the patient was normothermic with a temperature of 38.5°C. Other vital parameters were within normal limits, including a heart rate of 144 beats per minute, a respiratory rate of 36 breaths per minute, and a systolic blood pressure of 140 mmHg, measured using a Doppler ultrasonic flow detector. The mucous membranes were sticky, and skin turgor was mildly delayed, consistent with 5% dehydration. On blood examination, significant leukocytosis and elevated C-reactive protein (CRP) (169 mg/L; RI, 0–9 mg/L) were noted. Additionally, elevations in creatine kinase, bilirubin, alkaline phosphatase (ALKP), alanine aminotransferase (ALT), aspartate aminotransferase (AST), gamma-glutamyl transferase (GGT), and azotemia were observed. Serum symmetric dimethylarginine (SDMA) (57 μg/dL; RI, 0–14 μg/dL) was also increased. The complete blood count, serum biochemical, and venous blood gas results are summarized in Table 1. On coagulation examination, prothrombin time and activated partial thromboplastin time were both within the RI. However, the concentration of D-dimer was significantly elevated (4,360 ng/mL; reference interval, <250 ng/mL). Complete urinalysis obtained by cystocentesis revealed brown and turbid urine appearance with a specific gravity of 1.010, pH 7, 3+ protein, 3+ blood, and 2+ leukocyte. Hematuria was observed in the microscopic examination of the urine sediment, with no crystals or casts present. Thoracic and abdominal radiography revealed no remarkable findings. Abdominal ultrasonography was performed and revealed increased cortical echogenicity in both kidneys, hypoechoic echogenicity of the liver, hyperechoic pancreas with peripancreatic hyperechoic fat, gastric wall thickening, and decreased gastrointestinal motility. Severe multi-organ dysfunction, characterized by acute kidney injury [International Renal Interest Society acute kidney injury (AKI) grade 5], liver failure, and gastrointestinal failure, was suspected to result from direct toxins from massive paper wasp envenomation (4).

**Table 1:** Serial complete blood count, venous blood, and serum chemistry results from a dog with massive Paper wasp envenomation.

	**Reference interval**	**Presentation**	**Hour 20**	**Hour 44**
WBC (10^3^/μL)	6–17	32.89	13.76	28.31
HCT (%)	37–55	44.7	25.8	15.1
Platelet count (10^3^/μL)	200–500	291	115	55
Reticulocyte count (10^3^/μL)	10–110	162.9	77.4	75.4
pH	7.31–7.46	7.3	7.44	7.34
Na (mmol/L)	145–151	131	130	130
K (mmol/L)	3.9–5.1	5.4	6	7
Cl (mmol/L)	110–119	83	101	100
Calcium (mmol/L)	1.16–1.4	0.69	1.08	1.19
Glucose (mg/dL)	63–118	142.4	N/A	123.2
Albumin (g/dL)	2.6–4	3.15	N/A	1.69
ALT (U/L)	20–98	1,069	N/A	271
AST (U/L)	14–51	146	N/A	833
ALP (U/L)	17–111	692	N/A	545
GGT (U/L)	0–14	55	N/A	7
Total bilirubin (mg/dL)	0–0.2	9.79	5.07	4.86
Creatine kinase (U/L)	48–261	25,936	N/A	13,233
Triglyceride (mg/dL)	22–125	687.66	N/A	84.52
Cholesterol (mg/dL)	139–333	136.3	N/A	68

WBC, White blood cell; HCT, hematocrit; ALT, alanine transaminase; AST, aspartate aminotransferase; ALP, alkaline phosphatase; GGT, gamma-glutamyl transferase.

Initial therapeutics consisted of 10 ml/kg Ringer's lactate IV based on 70 mL/kg/day maintenance in addition to 5% dehydration aimed to be replaced over 8 h. Additionally, low dose epinephrine 0.01 mg/kg IV, dexamethasone 0.2 mg/kg IV, chlorpheniramine 0.2 mg/kg SC, maropitant 1 mg/kg SC, esomeprazole 1 mg/kg IV, ampicillin/sulbactam 12.5 mg/kg IV was added to the therapeutic plan. The dog was monitored in the intensive care unit after the placement of a urinary catheter. However, the dog remained anuric, with no urine output observed even after 8 h.

On day 1 of hospitalization, 8 h post-presentation, continuous venovenous hemodialysis was initiated using an automated renal replacement therapy and continuous fluid management unit. As the dog's condition continued deteriorate, diuretic therapy with furosemide or mannitol was not considered, and hemodialysis was prioritized for immediate management. Before use, an 8-Fr, 16-cm double lumen catheter was percutaneously placed in the right external jugular vein. The blood access lines and hemofilter were primed with heparinized saline. Unfractionated heparin was administered continuously as an anticoagulant dose ranging from 15 to 30 U/kg/h throughout the continuous renal replacement treatment (CRRT) procedure. A dialysate flow rate of 0–600 ml/h (0–123 ml/kg/h) and replacement rates of 50–100ml/h (10.2–20.5 ml/kg/h) and ultrafiltration rate of 20–50 ml/h (4.1–10.2 ml/kg/h) were utilized. There was no blood transfusion during the CRRT. A filtration fraction of 16.6%, measured Kt/V of 0.42, and calculated urea reduction ratio of 48.5% (5). Following the CRRT, BUN levels decreased from 181.7 mg/dL at admission to 93.5 mg/dL. Similarly, creatinine levels were reduced from 10.87 mg/dL to 4.04 mg/dL (Table 2).

**Table 2:** Results of serial renal biochemical panels taken at various time points following the massive paper wasp envenomation.

	**Reference interval**	**Presentation**	**Hour 9 (CRRT 1)**	**Hour 10 (CRRT 2)**	**Hour 11 (CRRT 3)**	**Hour 12 (CRRT 4)**	**Hour 20**	**Hour 44**
BUN (mg/dL)	10.1–31.9	181.7	140	116.8	106.5	93.5	107.8	112.9
Creatinine (mg/dL)	0.6–1.4	10.87	6.92	4.75	4.49	4.04	6.84	7.01
Phosphorus (mg/dL)	1.9–5.2	20.1	N/A	N/A	N/A	N/A	9.56	11.02

Continuous renal replacement therapy (CRRT) was initiated 8 h post-presentation and ended 4 h later.

By day 2 of hospitalization (20 h post-presentation), the dog remained persistently anuric, and its mental status deteriorated to a depressed state. The body weight increased from 4.88 kg to 5.5 kg, with the signs of overhydration including peripheral edema and increased skin turgor. The dog continued to show anorexia and dull mentation. The blood examination revealed that hyperbilirubinemia improved. However, azotemia progressed, with BUN at 107.8 mg/dL, creatinine at 6.84 mg/dL, and phosphorus at 9.56 mg/dL, compared to post-CRRT values. Venous blood gas analysis showed progressive hyperkalemia at 6 mmol/L. A second session of CRRT and blood transfusion were declined by the owner. On the second day, intravenous fluids were tapered and discontinued at 30 h post-presentation. Furosemide 2 mg/kg IV and Mannitol 1 g/kg IV were administered, but the patient remained anuric.

On day 3 of hospitalization, a venous blood gas revealed progressive hyperkalemia at 7 mmol/L. The chemistry panel showed a significant reduction in creatine kinase but progressive azotemia with BUN at 112.9 mg/dL, creatinine at 7.01 mg/dL, and phosphorus at 11.02 mg/dL. Administration of 1 ml/kg of 50% dextrose and 0.1 U/kg of regular insulin was initiated for the treatment of hyperkalemia. The patient progressively deteriorated, becoming stuporous, and showed bradycardia and hypotension at 48 h post-presentation, and cardiac arrest was observed. A post-mortem examination was declined by the owner.

## Discussion

This is the first case report describing the clinical characteristics of a dog envenomated by paper wasp (*Polistes rothneyi*). It confirms the occurrence of multi-organ lesions, including renal, hepatic, and gastrointestinal dysfunction, following massive paper wasp (*Polistes rothneyi*) envenomation. There are several reports of Hymenoptera stings in veterinary medicine, most of which involve bee stings, with other reports documenting stings by yellow jackets (*Vespula* spp) (6–10). Envenomation from paper wasps typically requires concern primarily for allergic reactions and cutaneous pain and inflammation which could result in fewer hospital visits in humans (11). Additionally, allergic reactions and fatalities are less common compared to yellowjacket stings, which may contribute to the limited information available on paper wasp envenomation (11).

The response to a Hymenoptera sting is classified into four categories: (1) local reactions, (2) uncomplicated allergic reactions, (3) anaphylactic reactions, and (4) direct systemic toxic reactions (12). During a sting, the wasp injects a proteinaceous liquid stored in its venom glands. The venom of Vespid wasps contains a variety of chemical constituents, including phospholipase A, antigen 5, hyaluronidase, acid phosphatase, biogenic amines, mast cell degranulating peptides, and kinins (1). These components can have myotoxic, hemolytic, neurotoxic, hepatotoxic, nephrotoxic, and vasodilatory effects (13).

A single wasp sting can cause an immunoglobulin E-mediated anaphylactic reaction, while mass envenomation from multiple stings can lead to systemic toxin-mediated cellular damage (14). In one study, acute kidney injury, rhabdomyolysis, hemolysis, liver injury, and coagulopathy were identified as the most frequent non-allergic manifestations of wasp stings (15). Additionally, high creatinine levels, presence of oliguria, anemia, and shock were recognized as risk factors for death (15). In this case, the dog was suspected to have direct systemic toxic reactions; however, the possibility of anaphylaxis could not be entirely ruled out, characterized by end-organ dysfunction and persistent gastrointestinal symptoms, including vomiting, melena, and facial swelling. Toxic reactions after Hymenoptera stings are uncommonly reported since most stinging events involve only one to a few stings, resulting in a small amount of toxin being injected into the body (2). However, in this study, the dog disturbed a shrub containing a paper wasp nest, leading to massive envenomation.

A severe acute kidney injury was observed in the dog at presentation, 3 days after massive envenomation. The pathophysiology of AKI following wasp envenomation is varied and includes secondary myoglobin release due to rhabdomyolysis, myoglobin-induced renal toxicity, intravascular hemolysis, direct nephrotoxicity from the wasp venom, allergic reactions, and inflammatory responses (16). The incidence of AKI following wasp envenomation is reported to be approximately 10 to 58% (17). In such patients, urinalysis often reveals various amounts of red and white blood cells with or without casts; the dog in our study showed similar results. Additionally, among those presenting with AKI, about 83% required dialysis (18). The dog in this study initially exhibited lethargy without significant symptoms after envenomation but later developed vomiting and diarrhea, which progressively worsened over time, eventually leading to an anuric state and hospitalization. It is crucial to recognize that even mild initial signs, such as lethargy, can progress to AKI. Additionally, the delay in presentation to the hospital may have contributed to the development of AKI and worsened the overall prognosis, highlighting the importance of timely medical intervention in similar cases.

Treatment for massive paper wasp envenomation was mainly supportive as there is no antivenom available for wasp stings (14). The patient underwent fluid resuscitation and received supportive treatment for toxin-related complications. A gastro-protectant, specifically a proton pump inhibitor, was administered, and neurokinin-1 receptor antagonist was prescribed to manage vomiting. Additionally, as anaphylaxis could not be ruled out, epinephrine was administered as the first-line drug, and antihistamines were given as adjunctive treatment (19). Continuous renal replacement therapy was initiated for the AKI, but the multi-organ dysfunction resulted in the patient remaining anuric and eventually cardiac arrest. Given that massive paper wasp envenomation can lead to systemic toxic reactions, including acute kidney failure, liver failure, and gastrointestinal failure, careful monitoring and fluid therapy could be considered as part of initial management. Although no established treatment guidelines exist for canine massive Hymenoptera envenomation, supportive care should be tailored to the patient's condition, and the potential benefits of additional treatments, such as anti-inflammatory or immunomodulatory therapies, warrant further investigation.

In humans, most victims of Hymenoptera stings sustain minor injuries and are treated as outpatients. In addition, if injuries are more serious, involving multiple stings by social wasps, patients tend to remain at medical facilities for ~4 days (3). In this case the dog experienced multiple stings but did not receive aggressive treatment initially. Despite later receiving intensive medication and dialysis, the patient eventually died.

## Conclusion

In conclusion, this is the first report describing clinical manifestation of massive paper wasp envenomation in veterinary medicine. Multiple organ dysfunction is a possible consequence of massive paper wasp envenomation. Given the potential risk of paper wasp envenomation in urban parks, increased environmental awareness is advised for dog owners. Both owners and clinicians should be cautious of wasp nests and take preventive measures. Timely and appropriate treatment should be considered from the outset to improve outcomes.

## Data Availability

The original contributions presented in the study are included in the article/supplementary material, further inquiries can be directed to the corresponding author.
